# A Performance Improvement Method for Low-Cost Land Vehicle GPS/MEMS-INS Attitude Determination

**DOI:** 10.3390/s150305722

**Published:** 2015-03-09

**Authors:** Li Cong, Ercui Li, Honglei Qin, Keck Voon Ling, Rui Xue

**Affiliations:** 1 School of Electronic and Information Engineering, Beihang University, 37 Xueyuan Road, Haidian District, Beijing 100191, China; E-Mails: buaa_liercui@163.com (E.L.); qhlmmm@sina.com (H.Q.); xuerui@buaa.edu.cn (R.X.); 2 School of Electrical and Electronic Engineering, Nanyang Technological University, 50 Nangyang Avenue 639798, Singapore; E-Mail: ekvling@ntu.edu.sg

**Keywords:** GPS, attitude determination, integer ambiguity resolution, CLAMBDA, MEMS-INS, ADOP, AFM

## Abstract

Global positioning system (GPS) technology is well suited for attitude determination. However, in land vehicle application, low-cost single frequency GPS receivers which have low measurement quality are often used, and external factors such as multipath and low satellite visibility in the densely built-up urban environment further degrade the quality of the GPS measurements. Due to the low-quality receivers used and the challenging urban environment, the success rate of the single epoch ambiguity resolution for dynamic attitude determination is usually quite low. In this paper, a micro-electro-mechanical system (MEMS)—inertial navigation system (INS)-aided ambiguity resolution method is proposed to improve the GPS attitude determination performance, which is particularly suitable for land vehicle attitude determination. First, the INS calculated baseline vector is augmented with the GPS carrier phase and code measurements. This improves the ambiguity dilution of precision (ADOP), resulting in better quality of the unconstrained float solution. Second, the undesirable float solutions caused by large measurement errors are further filtered and replaced using the INS-aided ambiguity function method (AFM). The fixed solutions are then obtained by the constrained least squares ambiguity decorrelation (CLAMBDA) algorithm. Finally, the GPS/MEMS-INS integration is realized by the use of a Kalman filter. Theoretical analysis of the ADOP is given and experimental results demonstrate that our proposed method can significantly improve the quality of the float ambiguity solution, leading to high success rate and better accuracy of attitude determination.

## 1. Introduction

In low-cost land vehicle navigation applications, not only are accurate position and velocity information required, but the navigation system also needs to provide accurate attitude [1,2,3,4]. Making use of the GPS signal for attitude determination has several advantages such as its small size, low cost, lack of cumulative errors and high accuracy. However, GPS attitude determination also has its own drawbacks. It is susceptible to the external environment and unstable in dynamic applications [4]. The traditional attitude determination method is to use INS. The drawback of INS is that the errors are accumulated over time especially for the low-cost MEMS-INS. If the GPS and MEMS-INS are combined effectively, the reliability and accuracy of attitude determination can be improved [5,6,7]. 

When a multiple-antenna GPS system is used for attitude determination, the challenge is how to determine the carrier phase integer ambiguity quickly, accurately and reliably. However, due to the loss of lock and noise disturbance, cycle slips often occur in land vehicle applications. Therefore, the single epoch method is usually adopted for dynamic attitude determination in such applications. This is because attitude determination in single epoch is insensitive to cycle slips. In addition, due to the low cost requirement, single frequency GPS receivers are widely used in this type of application, but the carrier phase and code (pseudorange) measurement qualities are both very low for low-cost GPS receivers, especially the code measurement [8]. Moreover, GPS signal is often blocked, attenuated or contaminated by multipath signals in urban areas. As pointed out in [9], these factors contribute to low ambiguity success rate, so the key to high accuracy GPS/MEMS-INS attitude determination is to improve the GPS ambiguity success rate. 

The ideas of using inertial sensors to improve GPS ambiguity success rate have been proposed recently [10,11,12,13,14,15]. These methods adopt MEMS inertial sensors to aid different ambiguity resolution methods. They make use of the MEMS-INS attitude information to reduce the integer ambiguity search region, thus improve the rapidity and reliability of ambiguity resolution. For example, the attitude information of rate gyros was employed in [10] to reduce the ambiguity search region to a small cube to improve the ambiguity resolution process which was based on the least squares ambiguity search technique. On the other hand, Zhu *et al.* [11] applied rate-gyro-constraints to filter the candidates in the ambiguity search stage, which can speed up the initialization of attitude parameters under dynamic circumstances. Eling [13] presented an instantaneous GNSS/MEMS attitude determination method which used AFM aided by MEMS to perform the single epoch ambiguity resolution. Roth *et al.* [14,15] explored a method that combined low-cost inertial and magnetic field sensors with a GNSS compass to provide a multi-sensor attitude system for portable, small-sized launcher applications. The method can improve the availability and reliability of pure GNSS attitude determination by using an extension of the LAMBDA method which accounts for baseline length and attitude constraints.

LAMBDA is popular for ambiguity resolution, since it is known to maximize the ambiguity success rate [9,16]. It has been used to obtain precise baseline estimations that are then used to extract the platform attitude [17,18,19,20]. However, the LAMBDA method does not include the prior knowledge of the length of the baseline, which is usually known in GPS attitude determination problems [9,21]. Therefore, the CLAMBDA method [22,23] is proposed for attitude determination by integrating the nonlinear baseline constraint into the ambiguity objective function. It further improves the success rate of attitude determination, especially for single frequency single epoch cases [24,25], but the CLAMBDA method is also challenged for the single frequency single epoch case in low-cost land vehicle applications, since the unconstrained float solution is usually of very low quality due to the poor quality GPS measurements, especially when the GPS signal is blocked or contaminated by multipath signals in urban areas, which results in decreased success rates [21,26]. Therefore, for such application, the quality of the float solution should be improved.

In this paper, a new method to improve the attitude determination performance by improving the quality of float ambiguity solution is proposed. First, the INS calculated baseline vector is augmented with GPS carrier phase and code measurements. The quality improvement of the float ambiguity solution is verified by theoretical analysis. Second, we use ADOP combined with the INS derived reference value to assess the quality of the float ambiguity solution. The undesirable float solution is replaced with the float solution obtained through the INS-aided AFM, where the INS attitude information is used to optimize the AFM search region. Moreover, some constraints are adopted for the selection of the correct float solution. Then, the fixed solution is obtained by the CLAMBDA method, and the GPS attitude measurement can be calculated. Finally, the GPS/MEMS-INS integrated filter is used to estimate the navigation errors and sensor errors of MEMS-INS, which can effectively improve the quality of the INS attitude measurement for the estimation of the float ambiguity solution. 

The rest of this paper is organized as follows: Section 2 gives an overview of the CLAMBDA method. Section 3 describes our improved attitude determination method for single frequency and single epoch. Section 4 introduces the GPS/MEMS-INS integrated filter algorithm. The experimental results of the new method are shown in Section 5. The final conclusions of this paper are given in Section 6.

## 2. GPS Attitude Determination with the CLAMBDA

For the GPS compass system, two antennas are utilized to provide the observability of yaw (or heading) and pitch (or elevation). The GPS compass model with the baseline constraint is presented as [9]:

(1) $$\begin{array}{l} E(y)=Aa+Bb,\left\|b\right\|=l,a\in Z^{n},b\in R^{3}\\ D(y)=Q_{y} \end{array}$$

where y is the given GPS data vector, and a and b are the ambiguity vector and baseline vector of order n and 3, respectively. E(⋅) and D(⋅) denote the expectation and dispersion operators, respectively. A and B are the given design matrices that link the data vector to the unknown ambiguity vector and baseline vector, respectively. The variance matrix of y is given by the positive definite matrix $Q_{y}.$ l is the baseline length, which is *a priori* given information.

If we apply the least squares (LS) estimation principle to solve for the unknown ambiguity vector a and baseline vector b , we need to solve the minimization problem:

(2) $$\underset{a\in Z^{n},\left\|b\right\|=l}{\text{min}}{\left\|y-Aa-Bb\right\|}_{Q_{y}}^{2}$$

Based on the orthogonal decomposition, the minimization problem of (2) can be formulated as:

(3) $$\underset{a\in Z^{n},\left\|b\right\|=l}{\text{min}}{\left\|y-Aa-Bb\right\|}_{Q_{y}}^{2}={\left\|\hat{e}\right\|}_{Q_{y}}^{2}+\underset{a\in Z^{n}}{\text{min}}({\left\|\hat{a}-a\right\|}_{Q_{\hat{a}}}^{2}+\underset{\left\|b\right\|=l}{\text{min}}{\left\|\hat{b}(a)-b\right\|}_{Q_{\hat{b}(a)}}^{2})$$

where $\hat{e}$ is the LS residual vector. $Q_{\hat{a}}$ and $Q_{\hat{b}(a)}$ are the variance-covariance (vc-) matrices of float ambiguity solution $\hat{a}$ and conditional baseline solution $\hat{b}(a)$ , respectively. 

The CLAMBDA method can minimize the following objective function to obtain the fixed ambiguity solution:

(4) $$\underset{a\in Z^{n}}{\text{min}}\left({\left\|\hat{a}-a\right\|}_{Q_{\hat{a}}}^{2}+{\left\|\hat{b}(a)-\overset{⌣}{b}(a)\right\|}_{Q_{\hat{b}(a)}}^{2}\right)\;\text{with}\;\overset{⌣}{b}(a)=\text{arg}\underset{\left\|b\right\|=l}{\text{min}}{\left\|\hat{b}(a)-b\right\|}_{Q_{\hat{b}(a)}}^{2}$$

Equation (4) has no analytic solution, and it should be solved using an efficient searching method. The detailed description of the searching method used here can be found in [23], which based on the “expansion” approach. After we obtain the fixed ambiguity solution, we can also obtain the baseline vector with respect to the local East-North-Up frame, which is used to calculate yaw and pitch.

## 3. Improved Attitude Determination Method for Single Frequency and Single Epoch

Our method is based on the orthogonal transformation model, which is a numerically stable approach [27]. For m=n+1 satellites in view, the orthogonal transformation model [28] of single difference carrier phase and pseudorange can be described as follows:

(5) $$\bar{P}y^{\text{ϕ}}=\bar{P}Eb-Fa+\bar{P}\nu^{\text{ϕ}},\;\bar{P}\nu^{\text{ϕ}}\sim N(0,2\sigma_{\text{ϕ}}^{2}I_{m-1})$$

(6) $$\bar{P}y^{\rho }=\bar{P}Eb+\bar{P}\mu^{\rho },\;\bar{P}\mu^{\rho }\sim N(0,2\sigma_{\rho }^{2}I_{m-1})$$

where $\bar{P}=[\frac{e}{\sqrt{m}},I_{m-1}-\frac{ee^{T}}{m-\sqrt{m}}],$ $F=I_{m-1}-\frac{ee^{T}}{m-\sqrt{m}},$ $e={\left(1,1,...,1\right)}_{(m-1)\times 1}^{T}.$ $y^{\text{ϕ}}$ is the single difference carrier phase and $y^{\rho }$ is the single difference pseudorange. b is the baseline vector and a is the double difference (DD) ambiguity. $\left\|b\right\|=l,a\in Z^{n},b\in R^{3}.$ $E=\lambda_{1}^{-1}{\left[\begin{array}{cccc} e_{1} & e_{2} & \cdots & e_{m} \end{array}\right]}^{T}$ contains the receiver-satellite unit line-of-sight vectors. $\lambda_{1}$ is the wavelength of L1 carrier. $\sigma_{\text{ϕ}}^{2}$ is the variance of carrier phase and $\sigma_{\rho }^{2}$ is the variance of pseudorange. $\nu^{\text{ϕ}}$ and $\mu^{\rho }$ are the single difference carrier phase and pseudorange noise vectors, respectively. The transformed noise vectors $\bar{P}\nu^{\text{ϕ}}$ and $\bar{P}\mu^{\rho }$ still follow the same distribution because orthogonal transformation will not change the statistical properties of white noise.

The first step of attitude determination with CLAMBDA is to obtain the float ambiguity solution $\hat{a}$ (which is the so-called unconstrained float ambiguity solution [21] in this paper) and its vc- matrix $Q_{\hat{a}}$ by the LS method [29]. $Q_{\hat{a}}$ contains all the information necessary to infer the quality of the float ambiguity solution. It can be seen that the smaller the $Q_{\hat{a}},$ the higher the quality of the float solution. In order to capture the main characteristics of $Q_{\hat{a}},$ the ADOP [30] is defined as:

(7) $$ADOP={\left(\sqrt{\left|Q_{\hat{a}}\right|}\right)}^{\frac{1}{n}}\;\;\;\left(\text{cycle}\right)$$

ADOP equals the geometric mean of the conditional standard deviations of $\hat{a}.$ Therefore, ADOP can be used for the quality assessment of the float solution. However, for the single frequency single epoch GPS attitude determination, if the quality of GPS measurements is very low, especially when GPS signal is blocked or contaminated by multipath signals, the float solution is usually of low quality. Although the CLAMBDA method can maximize the ambiguity success rate of attitude determination, the success rate is also not very high due to the low-quality float solution caused by poor quality GPS measurements. Therefore, we augment GPS measurements with MEMS-INS measurement to improve the quality of float solution. This is described in detail as follows.

### 3.1. Float Solution by GPS/INS Augmented Measurements

Assuming the expression of the INS calculated baseline vector in the local East-North-Up frame is $b_{I}={\left[\begin{array}{ccc} b_{IE} & b_{IN} & b_{IU} \end{array}\right]}^{T}$ , and the expression of baseline vector in the body frame is $b^{b}={\left[\begin{array}{ccc} b_{x}^{b} & b_{y}^{b} & b_{z}^{b} \end{array}\right]}^{T}$ . For the short baseline, the following equation is obtained:

(8) $$b_{I}=C_{b}^{n}b^{b}$$

(9) $$C_{b}^{n}=\left[\begin{array}{ccc} \text{sin}\psi_{I}\text{sin}\theta_{I}\text{sin}\gamma_{I}+\text{cos}\psi_{I}\text{cos}\gamma_{I} & \text{sin}\psi_{I}\text{cos}\theta_{I} & \text{cos}\psi_{I}\text{sin}\gamma_{I}-\text{sin}\psi_{I}\text{sin}\theta_{I}\text{cos}\gamma_{I}\\ \text{cos}\psi_{I}\text{sin}\theta_{I}\text{sin}\gamma_{I}-\text{sin}\psi_{I}\text{cos}\gamma_{I} & \text{cos}\psi_{I}\text{cos}\theta_{I} & -\text{sin}\psi_{I}\text{sin}\theta_{I}-\text{cos}\psi_{I}\text{sin}\theta_{I}\text{cos}\gamma_{I}\\ -\text{cos}\theta_{I}\text{sin}\gamma_{I} & \text{sin}\theta_{I} & \text{cos}\theta_{I}\text{cos}\gamma_{I} \end{array}\right]$$

where $C_{b}^{n}$ is the INS attitude matrix, which is orthogonal. $\psi_{I},\theta_{I},\gamma_{I}$ are the yaw, pitch and roll of INS, respectively. Assuming the vehicle is a rigid body, $b^{b}$ is unchanged, which can be obtained by accurate measurement. In order to unify the symbols and consider the measurement noise, we adjust Equation (8) as:

(10) $$b_{I}=C_{b}^{n}b^{b}=b+\varepsilon ,\varepsilon \sim N(0,\sigma_{I}^{2}I_{3})$$

where ε represents the measurement noise vector of the INS calculated baseline vector in the local frame and $\sigma_{I}^{2}$ is the corresponding noise variance. Actually, if the baseline vector is calculated by INS attitude, the entries of the measurement noise vector are correlated. Therefore, the measurement noise variance in Equation (10) is not diagonal. The detailed analysis is as follows: the main baseline vector in the local level frame is expressed as:

(11) $$b=\left[\begin{array}{c} l\text{cos}\theta sin\psi \\ l\text{cos}\theta \text{cos}\psi \\ l\text{sin}\theta \end{array}\right]$$

When the baseline length is fixed, the baseline vector can be expressed as the nonlinear function of yaw and pitch [31,32]. The nonlinear equations are linearized as:

(12) $$\begin{array}{l} \left[\begin{array}{c} \delta b_{IE}\\ \delta b_{IN}\\ \delta b_{IU} \end{array}\right]=\left[\begin{array}{cc} l\text{cos}\theta_{I}\text{cos}\psi_{I} & -l\text{sin}\theta_{I}\text{sin}\psi_{I}\\ -l\text{cos}\theta_{I}\text{sin}\psi_{I} & -l\text{sin}\theta_{I}\text{cos}\psi_{I}\\ 0 & l\text{cos}\theta_{I} \end{array}\right]\left[\begin{array}{c} \delta \psi_{I}\\ \delta \theta_{I} \end{array}\right]\\ =\left[\begin{array}{c} (l\text{cos}\theta_{I}\text{cos}\psi_{I})\delta \psi_{I}-(l\text{sin}\theta_{I}\text{sin}\psi_{I})\delta \theta_{I}\\ -(l\text{cos}\theta_{I}\text{sin}\psi_{I})\delta \psi_{I}-(l\text{sin}\theta_{I}\text{cos}\psi_{I})\delta \theta_{I}\\ (l\text{cos}\theta_{I})\delta \theta_{I} \end{array}\right] \end{array}$$

where the given Taylor point of expansion is at the INS attitude $\psi_{I},\theta_{I}$ . The second and higher order terms are neglected. The expression of the vc-matrix of the measurement noise vector is obtained as:

(13) $$VC(\varepsilon )=VC(\delta b_{I})=\left[\begin{array}{ccc} \sigma_{EE}^{2} & \sigma_{EN}^{2} & \sigma_{EU}^{2}\\ \sigma_{NE}^{2} & \sigma_{NN}^{2} & \sigma_{NU}^{2}\\ \sigma_{UE}^{2} & \sigma_{UN}^{2} & \sigma_{UU}^{2} \end{array}\right]$$

where:

$$\begin{array}{l} \sigma_{EE}^{2}={\left(l\text{cos}\theta_{I}\text{cos}\psi_{I}\right)}^{2}\sigma_{\psi_{I}}^{2}+{\left(l\text{sin}\theta_{I}\text{sin}\psi_{I}\right)}^{2}\sigma_{\theta_{I}}^{2}\\ \sigma_{NN}^{2}={\left(l\text{cos}\theta_{I}\text{sin}\psi_{I}\right)}^{2}\sigma_{\psi_{I}}^{2}+{\left(l\text{sin}\theta_{I}\text{cos}\psi_{I}\right)}^{2}\sigma_{\theta_{I}}^{2}\\ \sigma_{UU}^{2}={\left(l\text{cos}\theta_{I}\right)}^{2}\sigma_{\theta_{I}}^{2}\\ \sigma_{EN}^{2}=\sigma_{NE}^{2}=-{\left(l\text{cos}\theta_{I}\right)}^{2}\text{cos}\psi_{I}\text{sin}\psi_{I}\sigma_{\psi_{I}}^{2}+{\left(l\text{sin}\theta_{I}\right)}^{2}\text{cos}\psi_{I}\text{sin}\psi_{I}\sigma_{\theta_{I}}^{2}\\ \sigma_{EU}^{2}=\sigma_{UE}^{2}=-l^{2}\text{cos}\theta_{I}\text{sin}\theta_{I}\text{sin}\psi_{I}\sigma_{\theta_{I}}^{2}\\ \sigma_{NU}^{2}=\sigma_{UN}^{2}=-l^{2}\text{cos}\theta_{I}\text{sin}\theta_{I}\text{cos}\psi_{I}\sigma_{\theta_{I}}^{2} \end{array}$$

$\sigma_{\psi_{I}}^{2}$ and $\sigma_{\theta_{I}}^{2}$ are the measurement noise variances of the yaw and pitch of INS, respectively.

To simplify the calculation, the upper bounding approach [33,34] is adopted for the decorrelation of the measurement noise vector. A “more positive definite” diagonal matrix is selected as the upper bound of the vc-matrix of Equation (13). According to the positive definite matrix theory [33], the upper bound of the vc-matrix of Equation (13) should satisfy:

(14) $$\left[\begin{array}{ccc} \sigma_{I}^{2} & 0 & 0\\ 0 & \sigma_{I}^{2} & 0\\ 0 & 0 & \sigma_{I}^{2} \end{array}\right]-\left[\begin{array}{ccc} \sigma_{EE}^{2} & \sigma_{EN}^{2} & \sigma_{EU}^{2}\\ \sigma_{NE}^{2} & \sigma_{NN}^{2} & \sigma_{NU}^{2}\\ \sigma_{UE}^{2} & \sigma_{UN}^{2} & \sigma_{UU}^{2} \end{array}\right]>0$$

Therefore, $\sigma_{I}^{2}$ should be large enough to make sure above matrix is a positive definite matrix. It can be seen from Equation (13) that the upper bound satisfying Equation (14) can be conservatively selected by setting $\sigma_{I}^{2}=l^{2}\sigma_{\psi_{I}}^{2}+l^{2}\sigma_{\theta_{I}}^{2}$ , which is provable via determinants. After the vc-matrix of Equation (13) is replaced by above upper bound, the measurement noise vector satisfies $\varepsilon \sim N(0,\sigma_{I}^{2}I_{3}).$

Defining $\sigma_{1}=\sigma_{\text{ϕ}}/\sigma_{\rho }$ , $\sigma_{2}=\sigma_{\text{ϕ}}/\sigma_{I}$ , Equation (6) is multiplied by $\sigma_{1}$ , and Equation (10) is multiplied by $\sqrt{2}\sigma_{2}$ , then combining them with Equation (5) as:

(15) $$\left[\begin{array}{c} \bar{P}y^{\text{ϕ}}\\ \begin{array}{c} \sigma_{1}\bar{P}y^{\rho }\\ \sqrt{2}\sigma_{2}b_{I} \end{array} \end{array}\right]=\left[\begin{array}{c} \begin{array}{cc} \bar{P}E & -F \end{array}\\ \begin{array}{cc} \begin{array}{c} \sigma_{1}\bar{P}E\\ \sqrt{2}\sigma_{2}I_{3} \end{array} & \begin{array}{c} 0\\ 0 \end{array} \end{array} \end{array}\right]\left[\begin{array}{c} b\\ a \end{array}\right]+\left[\begin{array}{c} \bar{P}v^{\text{ϕ}}\\ \begin{array}{c} \sigma_{1}\bar{P}\mu^{\rho }\\ \sqrt{2}\sigma_{2}\varepsilon \end{array} \end{array}\right]$$

where $\left[\begin{array}{c} \bar{P}v^{\text{ϕ}}\\ \begin{array}{c} \sigma_{1}\bar{P}\mu^{\rho }\\ \sqrt{2}\sigma_{2}\varepsilon \end{array} \end{array}\right]\sim N(0,2\sigma_{\text{ϕ}}^{2}I_{2m+1})$ . Equation (15) can be expressed as follow:

(16) $$Y=Ax+w,w\sim N(0,Q_{Y})$$

where $Q_{Y}=2\sigma_{\text{ϕ}}^{2}I_{2m+1}$ . The state estimate obtained by the LS method is:

(17) $$\hat{x}={\left(A^{T}Q_{Y}^{-1}A\right)}^{-1}A^{T}Q_{Y}^{-1}Y$$

The vc-matrix of state estimate is:

(18) $$Q_{\hat{x}}=\left[\begin{array}{cc} Q_{\hat{b}\hat{b}} & Q_{\hat{b}\hat{a}}\\ Q_{\hat{a}\hat{b}} & Q_{\hat{a}\hat{a}} \end{array}\right]={\left(A^{T}Q_{Y}^{-1}A\right)}^{-1}$$

It is easy to prove that the quality of the float solution with the INS calculated baseline vector augmentation is higher than the quality of the float solution without the augmentation. 

Let:

(19) $$q=A^{T}Q_{Y}^{-1}A=\frac{1}{2\sigma_{\varphi }^{2}}A^{T}A=\frac{1}{2\sigma_{\varphi }^{2}}\left[\begin{array}{cc} {A^{\prime }}^{T} & G^{T} \end{array}\right]\left[\begin{array}{c} A^{\prime }\\ G \end{array}\right]=\frac{1}{2\sigma_{\varphi }^{2}}({A^{\prime }}^{T}A^{\prime }+G^{T}G)$$

where $A\prime =\left[\begin{array}{c} \begin{array}{cc} \bar{P}E & -F \end{array}\\ \begin{array}{cc} \sigma_{1}\bar{P}E & 0 \end{array} \end{array}\right]$ , $G=\left[\begin{array}{cc} \sqrt{2}\sigma_{2}I_{3} & 0_{3\times (m-1)} \end{array}\right]$ .

We can obtain the vc-matrix of state estimate:

(20) $$Q_{\hat{x}}=2\sigma_{\varphi }^{2}{\left({A^{\prime }}^{T}A^{\prime }+G^{T}G\right)}^{-1}$$

For the float solution without the augmentation, the vc-matrix of state estimate is:

(21) $$Q\prime_{\hat{x}}=2\sigma_{\varphi }^{2}{\left({A^{\prime }}^{T}A^{\prime }\right)}^{-1}$$

As $G^{T}G>0$ , it is easy to get $\left|Q_{\hat{x}}\right|<\left|{Q^{ʹ}}_{\hat{x}}\right|$ , and then $\left|Q_{\hat{a}\hat{a}}\right|<\left|{Q^{ʹ}}_{\hat{a}\hat{a}}\right|$ . It means that the ADOP with the INS calculated baseline vector augmentation is smaller than that without the augmentation. Therefore, the quality of float solution is improved by the GPS/INS augmented measurements. The improvement degree is related to the selection of $\sigma_{1}$ and $\sigma_{2}$ , which should be chosen reasonably. $\sigma_{1}$ and $\sigma_{2}$ are determined by $\sigma_{\text{ϕ}}$ , $\sigma_{\rho }$ and $\sigma_{I}$ . $\sigma_{\text{ϕ}}$ and $\sigma_{\rho }$ can be selected according to the measurement quality of the GPS receiver. $\sigma_{I}$ can be selected according to the measurement noise variances of MEM-INS attitude and the baseline length as $\sigma_{I}=\sqrt{l^{2}\sigma_{\psi_{I}}^{2}+l^{2}\sigma_{\theta_{I}}^{2}}$ .

It also can be seen from Equations (15), (20) and (21) that more accurate ambiguity float solution can be obtained as long as the number of tracked satellites is not less than two satellites, which is more important for the land vehicle attitude determination.

### 3.2. Quality Assessment of Float Solution

Theoretical analysis results of the ADOP in Section 3.1 demonstrate that better quality of the float solution can be obtained by the GPS/INS augmented measurements. However, low-cost GPS receivers may produce abnormal measurement errors (especially pseudorange errors), which are mainly caused by signal attenuation or multipath in the urban area [35]. This results in part of the float solutions still having large deviations. 

In order to reduce the effect of large outliers in pseudorange on the float ambiguity solution, a simple quality control (QC) method is adopted here, which is based on the residual chi-square test [6,10]. As the float solution is calculated by LSQ method, the key issue is how to construct the residual vector. Here, we use the INS calculated baseline vector to calculate the predicted psedorange measurements, and obtain the residual vector as:

(22) $$v_{\rho }=\bar{P}y^{\rho }-\bar{P}Eb_{I}=\bar{P}Eb-\bar{P}Eb_{I}+\bar{P}\mu^{\rho }=\bar{P}E(b-b_{I})+\bar{P}\mu^{\rho }$$

where the variance of the residual vector is:

$$Q_{v_{\rho }}=\sigma_{I}^{2}\bar{P}EE^{T}{\bar{P}}^{T}+2\sigma_{\rho }^{2}I_{m-1}$$

Then we can perform the residual chi-square test [6,10] on psedorange measurements. In order to detect the large outliers in pseudorange measurements correctly, $\sigma_{I}^{2}$ should be conservatively selected to describe the error of the INS calculated baseline vector.

However, undetected measurement errors may still result in some low-quality float ambiguity solutions. In order to achieve high success rate, these low-quality float solutions need to be filtered and improved. So the quality of the float solution estimated by Equation (17) should be assessed first. 

Since the attitude errors of MEMS-INS are estimated and corrected by the GPS/MEMS-INS integrated filter in real time, which will be introduced in Section 4, the errors usually are not very large [13,20]. Thus the float solution calculated by MEMS-INS attitude can be regarded as the reference. The error range of the float solution is related with the GPS/INS augmented measurements. So the ADOP calculated by the augmented measurement equations can be used for the quantitative description of the error range. Therefore, the range of the float solution can be determined by the MEMS-INS attitude and the ADOP.

First, the INS attitude calculated baseline vector ***b****I* in the local frame can be obtained by Equation (8). Then according to the carrier phase measurement Equation (5), the INS calculated float ambiguity solution ${\hat{a}}_{I}$ can be solved as follows:

(23) $${\hat{a}}_{I}=-F^{-1}\bar{P}(y^{\text{ϕ}}-Eb_{I})$$

Second, the ADOP can be calculated with Equations (7) and (18). Then, according to the 3σ principle, the float solution lies within the 3σ bound with a high probability, thus the range of each entry of DD float ambiguity solution $\hat{a}$ is chosen as:

(24) $${\hat{a}}_{Ii}-3\cdot \text{ADOP}\leq {\hat{a}}_{i}\leq {\hat{a}}_{Ii}+3\cdot \text{ADOP},\;i=1,\ldots ,m-1$$

If one entry of the float solution estimated by Equation (17) does not satisfy Equation (24), the float solution is marked as low-quality.

### 3.3. Float Solution with INS-Aided AFM

For the low-quality float solution filtered by Equation (24), we will replace it with the float solution resolved by the INS-aided AFM. The basic idea of AFM is that for the correct attitude, the value of the adaptive function should theoretically have the value 1 as the maximum, since the ambiguity is integer. The maximum of the adaptive function ideally leads to the correct ambiguity [36]. AFM does not use pseudorange measurement to calculate the float ambiguity solution, so it rejects the influence of bad pseudorange, which is an advantage of the AFM method. MEMS-INS attitude can be used to determine approximate attitude to reduce the size of the search space [13]. It can improve the computational efficiency and reliability of the ambiguity resolution. When searching in the space constrained by INS attitude, the correct ambiguity is always contained, so a high-quality float solution can be obtained. 

Given the pitch $\theta_{k}$ , yaw $\psi_{k}$ and baseline length l , the main baseline vector ***b****k* of the vehicle can be calculated by Equation (11), then the float solution ${\hat{a}}_{k}$ can be calculated by Equation (23). Accordingly, the adaptive function of the float solution can be expressed as:

(25) $$F\left(\theta_{k},\psi_{k}\right)=\frac{1}{m-1}\sum_{i=1}^{m-1}\text{cos}(2\pi {\hat{a}}_{ki})$$

The search space is determined by MEMS-INS attitude. $\theta_{I},\psi_{I}$ are the pitch and yaw of MEMS-INS, respectively. The corresponding error ranges are $\Delta \theta_{I}$ (>0) and $\Delta \psi_{I}$ (>0). Then the search space of pitch is $\left(\theta_{I}-\Delta \theta_{I},\theta_{I}+\Delta \theta_{I}\right)$ , and that of yaw is $\left(\psi_{I}-\Delta \psi_{I},\psi_{I}+\Delta \psi_{I}\right)$ . The limited space efficiently omits many false attitude candidates. After proper selection of the search step $\left(\tau_{\theta },\tau_{\psi }\right)$ , all possible attitude candidates are substituted into Equation (23) to calculate the float solution candidates. The candidate corresponding to the maximum value of adaptive function is the correct solution. 

However, despite the search space reduction, several candidates of similar magnitudes may still exist in the adaptive function. Therefore, in order to identify the most likely correct candidate, we need the validation procedure with some constrain conditions. Before the validation, for each float solution candidate, the fixed solution is obtained through rounding. The corresponding baseline vector ${\hat{b}}_{k}$ can be estimated by LS method, then the attitude $\left({\hat{\theta }}_{k},{\hat{\psi }}_{k}\right)$ can be calculated. 

The validation procedure is divided into three consecutive steps: 

(1) Baseline length verification

For the correct float solution candidate, the error between the estimated and known baseline length is very small, so if the estimated baseline length satisfies $l-\delta l\leq \left|{\hat{b}}_{k}\right|\leq l+\delta l$ , where δl=0.01l , the candidate will be sent to next step.

(2) Attitude verification

For the candidate ${\hat{a}}_{k}$ corresponding to $\left(\theta_{k},\psi_{k}\right)$ , if $\left(\theta_{k},\psi_{k}\right)$ is very close to the real pitch and yaw, the calculated $\left({\hat{\theta }}_{k},{\hat{\psi }}_{k}\right)$ should not be far from $\left(\theta_{k},\psi_{k}\right)$ . Thus if $\left|{\hat{\theta }}_{k}-\theta_{k}\right|$ and $\left|{\hat{\psi }}_{k}-\psi_{k}\right|$ are smaller than the thresholds, the candidate will be sent to next step. Here, the thresholds can be conservatively set to the search steps $\left(\tau_{\theta },\tau_{\psi }\right)$ .

(3) The residual verification

When the fixed solution and the estimated baseline vector of each candidate are substituted back to Equation (5), the corresponding residual can be obtained. The correct candidate will make the residual reach the minimum. Thus the remaining candidates are sorted according to the ascending order of their residuals. The float solution of the maximum value of adaptive function is selected from the first k candidates. The k can be set according to the number of remaining candidates. If the validation procedure returns empty, increase the δl , and repeat the whole validation procedure from step (1) to step (3).

### 3.4. The Implementation of the Proposed Method

**Figure 1:**
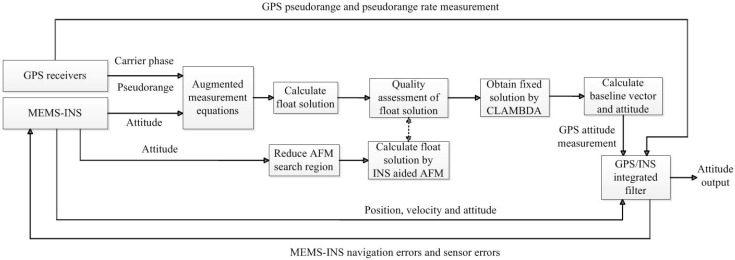
Flow diagram of the whole method.

The flow diagram of the method is shown in Figure 1. First, the INS calculated baseline vector is augmented with the GPS carrier phase and code measurements. Second, according to the result of quality assessment, the undesirable ambiguity float solution is replaced with the float solution obtained through INS aided AFM, where the INS attitude is used to reduce the AFM search region. Then, the fixed solution is obtained by the CLAMBDA method, which is used to calculate GPS attitude measurement. Finally, in order to effectively control the quality of the INS attitude measurement for the estimation of the float ambiguity solution, the GPS/INS integrated filter is used to estimate the navigation errors and sensor errors of MEMS-INS, which will be introduced in the next section. 

The proposed method can significantly improve the quality of the float ambiguity solution. It can not only improve the success rate of attitude determination, but also the computational efficiency. Since when the float solution is accurate, the search time of the fixed solution by CLAMBDA is always very short.

## 4. GPS/MEMS-INS Integrated Filter

After the attitude determination using MEMS-INS aided multiple-antenna GPS, GPS/MEMS-INS integration is also necessary. The integrated Kalman filter estimates the navigation errors and sensor errors using the GPS pseudorange, pseudorange rate and attitude measurements.

### 4.1. State Equations of the Integrated Filter

The states of integrated filter are given by:

(26) $$X={\left[X_{I}^{T}\;\;\;\delta \omega^{T}\;\;\;\;\delta f^{T}\;\;\delta t_{u}\;\;\delta tr_{u}\right]}_{17\times 1}^{T}$$

where $X_{I}={\left[\begin{array}{ccccccccc} \delta \theta & \delta \gamma & \delta \psi & \delta L & \delta \lambda & \delta h & \delta V_{e} & \delta V_{n} & \delta V_{u} \end{array}\right]}_{9\times 1}^{T}$ are the error states of INS, δθ , δγ , δψ are the pitch, roll and yaw errors, respectively; δL , δλ , δh are the longitude, latitude and height errors, respectively; $\delta V_{e}$ , $\delta V_{n}$ , $\delta V_{u}$ are the velocity errors of east, north and up, respectively. δω and δf are gyro and accelerometer error vectors, respectively. $\delta t_{u}$ and $\delta tr_{u}$ are the clock and frequency errors of GPS receiver.

Continuous state equations are described by:

(27) $$\dot{X}=FX+GW$$

$$\left[\begin{array}{c} {\dot{X}}_{I}\\ \delta \dot{\omega }\\ \delta \dot{f}\\ \dot{\delta }t_{u}\\ \delta \dot{t}r_{u} \end{array}\right]=\left[\begin{array}{ccccc} F_{11}^{9\times 9} & F_{12}^{9\times 3} & F_{13}^{9\times 3} & 0_{9\times 1} & 0_{9\times 1}\\ 0_{3\times 9} & \text{-(1/}\beta_{\omega }\;)I_{3\times 3} & 0_{3\times 3} & 0_{3\times 1} & 0_{3\times 1}\\ 0_{3\times 9} & 0_{3\times 3} & \text{-(1/}\beta_{f}\;)I_{3\times 3} & 0_{3\times 1} & 0_{3\times 1}\\ 0_{1\times 9} & 0_{1\times 3} & 0_{1\times 3} & 0 & 1\\ 0_{1\times 9} & 0_{1\times 3} & 0_{1\times 3} & 0 & \text{-1/}\beta_{tr} \end{array}\right]\left[\begin{array}{c} {\dot{X}}_{I}\\ \delta \dot{\omega }\\ \delta \dot{f}\\ \dot{\delta }t_{u}\\ \delta \dot{t}r_{u} \end{array}\right]+\left[\begin{array}{ccc} 0_{9\times 3} & 0_{9\times 3} & 0\\ I_{3\times 3} & 0_{3\times 3} & 0\\ 0_{3\times 3} & I_{3\times 3} & 0\\ 0_{1\times 3} & 0_{1\times 3} & 0\\ 0_{1\times 3} & 0_{1\times 3} & 1 \end{array}\right]\left[\begin{array}{c} W_{\omega }\\ W_{f}\\ w_{tr} \end{array}\right]$$

The INS error state equation of [6] is adopted here, the sub-matrices $F_{11}^{\;9\times 9}$ , $F_{12}^{\;9\times 3}$ and $F_{13}^{\;9\times 3}$ are given by:

$$\begin{array}{c} F_{11}^{9\times 9}=\left[\begin{array}{ccccccccc} 0 & \Omega \text{sin}\lambda +\frac{V_{e}\text{tan}\lambda }{R_{t}+h} & -\Omega \text{cos}\lambda -\frac{V_{e}}{R_{t}+h} & 0 & 0 & 0 & 0 & -\frac{1}{R_{m}+h} & 0\\ -\Omega \text{sin}\lambda -\frac{V_{e}\text{tan}\lambda }{R_{t}+h} & 0 & -\frac{V_{n}}{R_{m}+h} & 0 & 0 & 0 & \frac{1}{R_{t}+h} & 0 & 0\\ \Omega \text{cos}\lambda +\frac{V_{e}}{R_{t}+h} & \frac{V_{n}}{R_{m}+h} & 0 & 0 & 0 & 0 & \frac{\text{tan}\lambda }{R_{t}+h} & 0 & 0\\ 0 & 0 & 0 & 0 & \frac{V_{e}\text{tan}\lambda }{(R_{t}+h)cos\lambda } & -\frac{V_{e}}{{\left(R_{t}+h\right)}^{2}cos\lambda } & \frac{1}{(R_{t}+h)cos\lambda } & 0 & 0\\ 0 & 0 & 0 & 0 & 0 & -\frac{V_{n}}{{\left(R_{m}+h\right)}^{2}} & 0 & \frac{1}{R_{m}+h} & 0\\ 0 & 0 & 0 & 0 & 0 & 0 & 0 & 0 & 1\\ 0 & -f_{U} & f_{N} & 0 & 0 & 0 & 0 & 2\Omega \text{sin}\lambda +\frac{V_{e}\text{tan}\lambda }{R_{t}+h} & -2\Omega \text{cos}\lambda -\frac{V_{e}}{R_{t}+h}\\ f_{U} & 0 & -f_{E} & 0 & 0 & 0 & -2\Omega \text{sin}\lambda -\frac{V_{e}\text{tan}\lambda }{R_{t}+h} & 0 & -\frac{V_{n}}{R_{m}+h}\\ -f_{N} & f_{E} & 0 & 0 & 0 & \frac{2g_{0}}{R_{0}+h} & 2\Omega \text{cos}\lambda +\frac{V_{e}}{R_{t}+h} & \frac{V_{n}}{R_{m}+h} & 0 \end{array}\right]\\ F_{12}^{9\times 3}=\left[\begin{array}{c} C_{b}^{n}\\ 0_{3\times 3}\\ 0_{3\times 3} \end{array}\right].\;F_{13}^{9\times 3}=\left[\begin{array}{c} 0_{3\times 3}\\ 0_{3\times 3}\\ C_{b}^{n} \end{array}\right] \end{array}$$

where $R_{t}$ , $R_{m}$ and $R_{0}$ are the tangential radius, the meridional radius and the mean radius of the Earth, respectively; the symbols $g_{0}$ and Ω are local gravity and the Earth’s rotation rate, respectively. The gyro, accelerometer, and the receiver frequency errors are modeled as first-order Markov processes, and $\beta_{\omega }$ , $\beta_{f}$ and $\beta_{tr}$ are the corresponding correlation coefficients.

### 4.2. Measurement Equations of the Integrated Filter

The measurements of the integrated filter are the difference of pseudorange, pseudorange rate and attitude between GPS and MEMS-INS, which are given by:

(28) $$Z_{\rho }=\left[\begin{array}{c} \rho_{I1}-\rho_{G1}\\ \begin{array}{c} \rho_{I2}-\rho_{G2}\\ \vdots \end{array}\\ \rho_{Im}-\rho_{Gm} \end{array}\right]\;\;\;Z_{\dot{\rho }}=\left[\begin{array}{c} {\dot{\rho }}_{I1}-{\dot{\rho }}_{G1}\\ \begin{array}{c} {\dot{\rho }}_{I2}-{\dot{\rho }}_{G2}\\ \vdots \end{array}\\ {\dot{\rho }}_{Im}-{\dot{\rho }}_{Gm} \end{array}\right]\;\;\;Z_{a}=\left[\begin{array}{c} \theta_{I}-\theta_{G}\\ \gamma_{I}-\gamma_{G}\\ \psi_{I}-\psi_{G} \end{array}\right]$$

The measurement equation of pseudorange is described by [37]:

(29) $$Z_{\rho }=H_{\rho }X+V_{\rho }$$

where $H_{\rho }={\left[\begin{array}{ccc} H_{\rho 1}^{T} & \cdots & H_{\rho m}^{T} \end{array}\right]}^{T}$ , $H_{\rho j}={\left[\begin{array}{ccccccccc} 0_{1\times 3} & a_{j1} & a_{j2} & a_{j3} & 0_{1\times 3} & 0_{1\times 3} & 0_{1\times 3} & 1 & 0 \end{array}\right]}_{1\times 17}$ , $a_{j1}=-(R_{t}+h)\left[e_{j1}\text{cos}L\text{sin}\lambda -e_{j2}\text{cos}L\text{cos}\lambda \right]$ $a_{j2}=(R_{t}+h)\left[-e_{j1}\text{sin}L\text{cos}\lambda -e_{j2}\text{sin}L\text{sin}\lambda \right]+[R_{t}(1-e^{2})+h]e_{j3}\text{cos}L$ $a_{j3}=e_{j1}\text{cos}L\text{cos}\lambda +e_{j2}\text{cos}L\text{sin}\lambda +e_{j3}\text{sin}L$ $e_{j}={\left[\begin{array}{ccc} e_{j1} & e_{j2} & e_{j3} \end{array}\right]}^{T}$ is the unit vector heading to satellite j .

The measurement equation of pseudorange rate is described by:

(30) $$Z_{\dot{\rho }}=H_{\dot{\rho }}X+V_{\dot{\rho }}$$

where $H_{\dot{\rho }}={\left[\begin{array}{ccc} H_{\dot{\rho }1}^{T} & \cdots & H_{\dot{\rho }m}^{T} \end{array}\right]}^{T}$ , $H_{\dot{\rho }j}={\left[\begin{array}{ccccccccc} 0_{1\times 3} & 0_{1\times 3} & b_{j1} & b_{j2} & b_{j3} & 0_{1\times 3} & 0_{1\times 3} & 0 & 1 \end{array}\right]}_{1\times 17}$ , $b_{j1}=-e_{j1}\text{cos}\lambda \text{sin}L-e_{j2}\text{sin}L\text{sin}\lambda +e_{j3}\text{cos}L$ , $b_{j2}=-e_{j1}\text{sin}\lambda +e_{j2}\text{cos}\lambda$ , $b_{j3}=e_{j1}\text{cos}L\text{cos}\lambda +e_{j2}\text{cos}L\text{sin}\lambda +e_{j3}\text{sin}L$

The measurement equation of attitude is described by:

(31) $$Z_{a}=\left[\begin{array}{c} \theta_{I}-\theta_{G}\\ \gamma_{I}-\gamma_{G}\\ \psi_{I}-\psi_{G} \end{array}\right]=\left[\begin{array}{c} \delta \theta \\ \delta \gamma \\ \delta \psi \end{array}\right]+\left[\begin{array}{c} V_{\theta }\\ V_{\gamma }\\ V_{\psi } \end{array}\right]=H_{a}X+V_{a}$$

where $H_{a}={\left[\begin{array}{cc} I_{3\times 3} & 0_{3\times 14} \end{array}\right]}_{3\times 17}$ .

Then the measurement equations of the integrated filter can be obtained:

(32) $$Z=\left[\begin{array}{c} H_{\rho }\\ \begin{array}{c} H_{\dot{\rho }}\\ H_{a} \end{array} \end{array}\right]X+\left[\begin{array}{c} V_{\rho }\\ \begin{array}{c} V_{\dot{\rho }}\\ V_{a} \end{array} \end{array}\right]=HX+V$$

Based on the discrete forms of Equations (27) and (32), we can implement Kalman filter algorithm to estimate the MEMS-INS errors in real time. The Kalman filter algorithm is composed of prediction and measurement update [6]. Using the error estimates of the integrated filter to correct MEMS-INS errors, we can obtain the final attitude determination results. After feedback correction to the MEMS-INS navigation processing, MEMS-INS can provide better attitude measurement for the estimation of the float ambiguity solution.

## 5. Experiment Test Results

**Figure 2:**
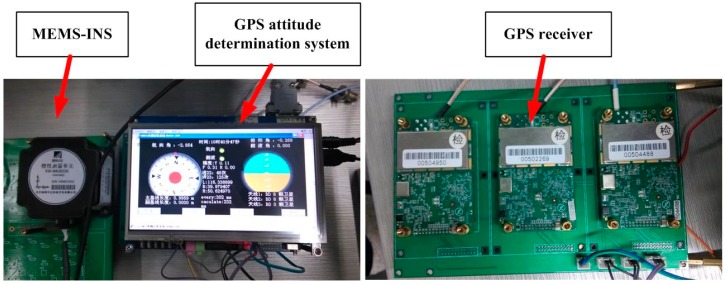
The test GPS/MEMS-INS attitude determination system.

Field tests in an urban area were conducted to verify the performance of the GPS/MEMS-INS integrated attitude determination system. The test system is shown in Figure 2. It consists of a MEMS-INS and the GPS attitude determination system developed by our research group. The GPS attitude determination system includes three receivers, an interface unit, a navigation processing unit, and a display unit. The interface unit includes a serial port for MEMS-INS. The system can work in real-time processing mode or data collection mode. 

The receivers used here are the SUPERSTAR II, which is the NovAtel 12 channels single frequency GPS receiver with 1 Hz output. Its code measurement precision is 0.75 m RMS. The difference carrier phase measurement precision is 0.01 m RMS. The MEMS-INS is the XW-IMU5220 from Beijing Starneto Technology Co. Ltd. (Beijing, China). The bias stability of gyro and accelerometer are 0.02°/s and 8 mg, respectively. The output frequency is 100 Hz.

### 5.1. Test Settings

**Figure 3:**
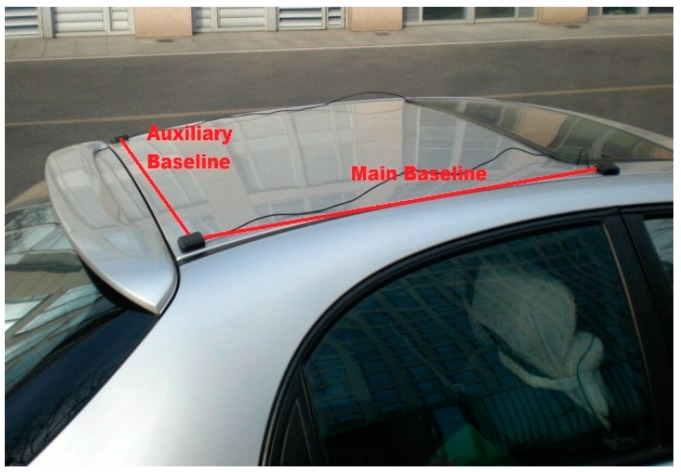
Experimental setup.

**Figure 4:**
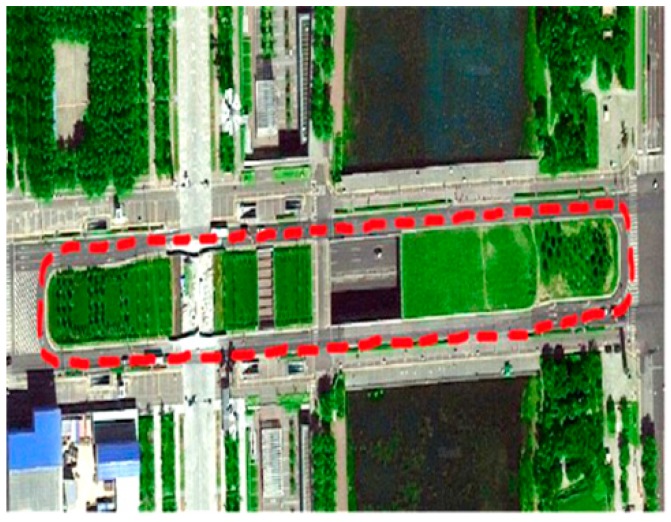
Experimental site.

**Figure 5:**
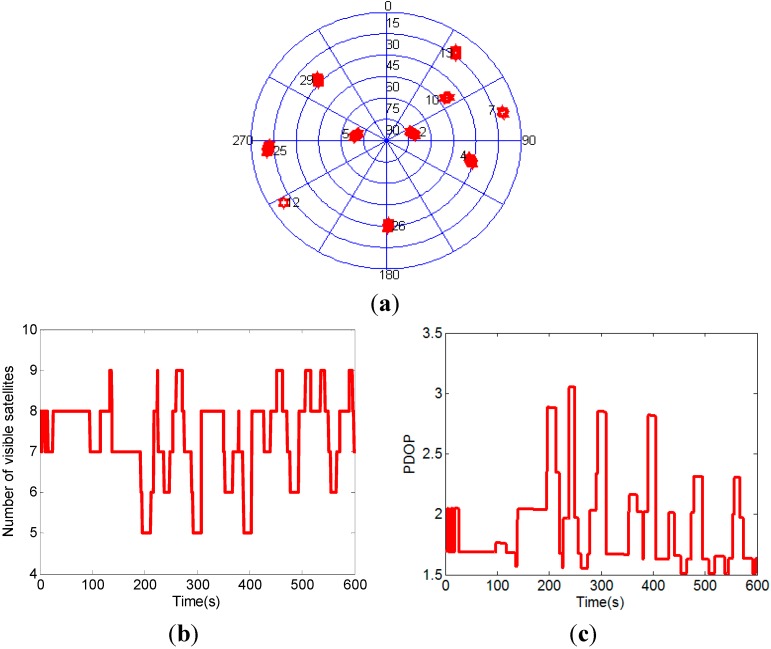
The distribution (**a**), number (**b**) and PDOP (**c**) of actual visible GPS satellites.

The experimental setup of the attitude determination system is shown in Figure 3. Two GPS baselines were mounted on the roof of the car. The main baseline which is used to determine the vehicle pitch and yaw comprises two antennas aligned in the driving direction. The auxiliary baseline which is used to determine the roll is at right-angled to the main baseline. The main baseline length is 1.0 m and the auxiliary baseline length is 0.9 m. The attitude determination system was mounted in the car trunk, and the measurement axes of MEMS-INS were corresponded with the baseline directions. The experimental site is on the east side of DaTun Road, Beijing Olympic Park, which is shown in Figure 4. The car moves along a long and narrow rectangular block for about five laps and both ends of the rectangle block are arc-shaped. The experimental time is about ten minutes. The distribution, number and PDOP of actual visible GPS satellites are shown in Figure 5. The number of visible satellites changes frequently due to the blockage of surrounding buildings. 

**Figure 6:**
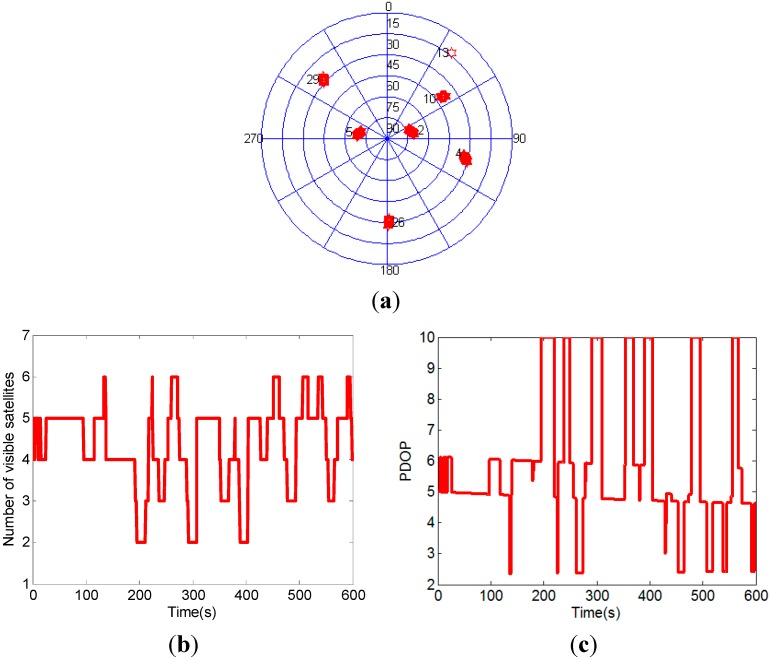
The distribution (**a**), number (**b**) and PDOP (**c**) of the visible GPS satellites after three satellites with lowest elevation angles removed.

In order to further test the performance of the attitude determination system, three satellites are removed to create a GPS challenged environment, *i.e.*, three satellites which have the lowest elevation angles are regarded as invisible. Figure 6 shows the distribution, number and PDOP of the visible GPS satellites after three satellites with lowest elevation angles removed. In this GPS challenged environment, the scenario with less than four visible satellites frequently occurs.

### 5.2. Test Results

The results of the proposed MEMS-INS aided GPS attitude determination method are compared with the unaided method. The performance improvements are verified through examing the quality of float solution, success rate and attitude accuracy. In the following, the default environment is the actual GPS environment shown in Figure 5, and the default baseline is the main baseline.

(1) The quality of float solution verification

**Figure 7:**
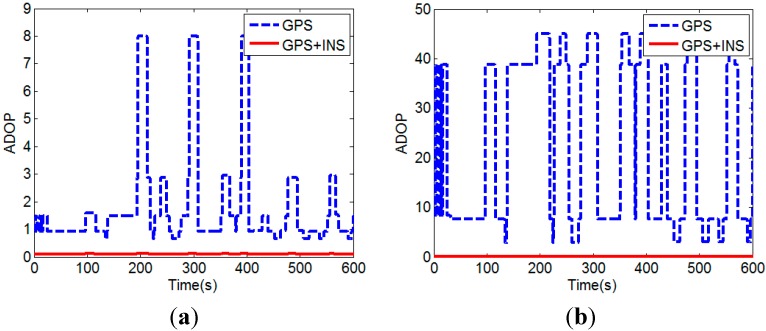
(**a**) Comparison of ADOPs with and without the INS calculated baseline vector augmentation for actual visible satellites; (**b**) Comparison of ADOPs with and without the INS baseline vector augmentation for the visible satellites after three satellites with lowest elevation angles removed.

We use the ADOP to assess the quality of the float ambiguity solution. Figure 7 compares the ADOPs with the INS calculated baseline vector augmentation (GPS + INS) and without the augmentation (GPS), for actual visible satellites (All) and the visible satellites after three satellites with lowest elevation angles removed (‒3), respectively. 

**Figure 8:**
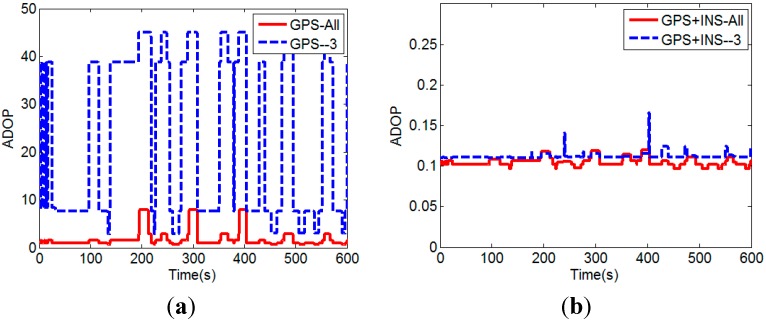
(**a**) ADOPs without the INS calculated baseline vector augmentation; (**b**) ADOPs with the INS calculated baseline vector augmentation.

It can be seen that the quality of the float solution is improved (*i.e.*, smaller ADOP) when the INS calculated baseline vector is augmented with the GPS carrier phase and code measurements. Since the precisions of the INS calculated baseline vector and GPS carrier phase are higher than that of the pseudorange, the contribution of GPS pseudorange is decreased after using the INS calculated baseline vector augmentation. The improvement is especially significant when additional three satellites with lowest elevation angles are removed. In other words, with the INS calculated baseline vector augmentation, the ADOP is less affected by the number of visible satellites. This can be seen clearly in Figure 8. 

**Figure 9:**
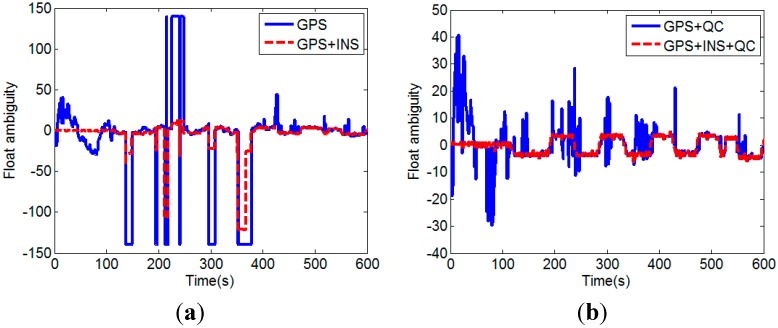
DD float ambiguity solutions of No.5 satellite without QC on pseudorange (**a**) and with QC on pseudorange (**b**).

It demonstrates that high-quality float solution can be obtained by using GPS/INS augmented measurements, even if the number of visible satellites is less than four. Since the float solution cannot be calculated using only GPS measurements when the number of visible satellites is less than four, the ADOP value is set to 45 for this situation to draw the figures. We next investigate the float solutions of our proposed method. In the test runs, satellite No. 2 and No. 5 were always visible. Satellite No. 2 had the highest elevation angle and it was selected as the reference satellite. Figure 9 shows the DD ambiguity float solution of satellite No. 5.

**Figure 10:**
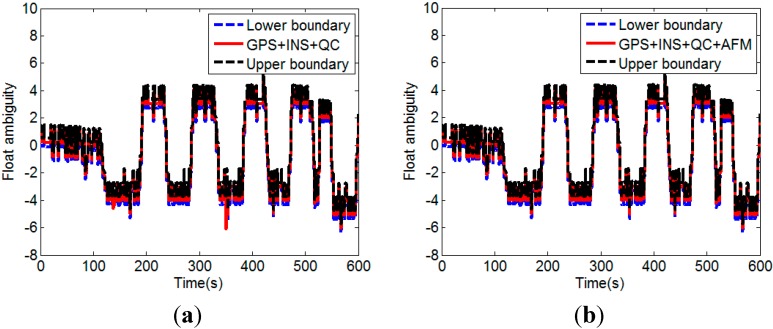
Float solutions before (**a**) and after (**b**) the replacement of the low-quality float solutions with the float solutions resolved by the INS aided AFM.

It can be seen that the float solutions without the INS calculated baseline augmentation is of very poor quality, especially for the case without QC on pseudorange. Large deviations in the float ambiguity can be effectively eliminated by the QC on pseudorange. Incorporating the INS calculated baseline vector improves the float solution significantly. Figure 10a shows the float solutions with the INS calculated baseline augmentation and the QC on pseudorange (GPS + INS + QC). It can been seen that most of the float solutions are inside the range determined by (24). However, there are still several float solutions that lie outside the bounds. These low-quality float solutions are replaced with the float solutions resolved by the INS aided AFM described in Section 3.3. Figure 10b shows the float solutions after the replacement (GPS + INS + QC + AFM).

**Figure 11:**
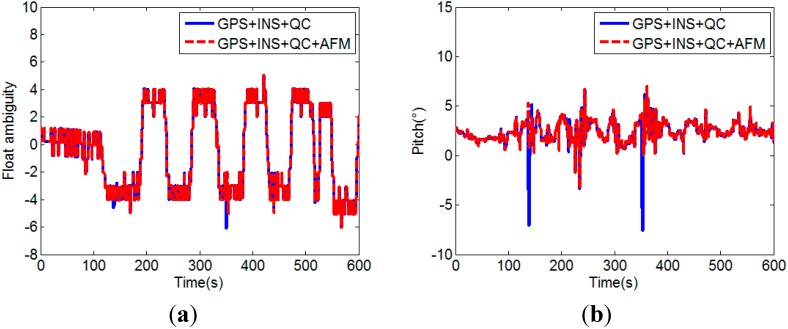
(**a**) Comparison of float solutions with and without the low-quality float solutions replacement; (**b**) Comparison of pitches calculated with and without the low-quality float solutions replacement.

From Figure 10b, it can been seen that 100% of the replaced float solutions are inside the range determined by Equation (24). Figure 11 shows the attitude determination results corresponding to the float solutions with and without the low-quality float solutions replacement. It clearly indicates that when the low-quality float solutions are used, the calculated attitudes are always wrong. The quality improvement of float solution is an important factor for successful attitude determination with CLAMBDA. 

The test results above demonstrate that the quality of float solution can be efficiently improved by the proposed MEMS-INS aided GPS attitude determination method, which including the GPS/INS augmented measurements, the quality assessment of float solution, and low-quality float solution replacement with INS aided AFM. 

(2) Success rate verification

**Figure 12:**
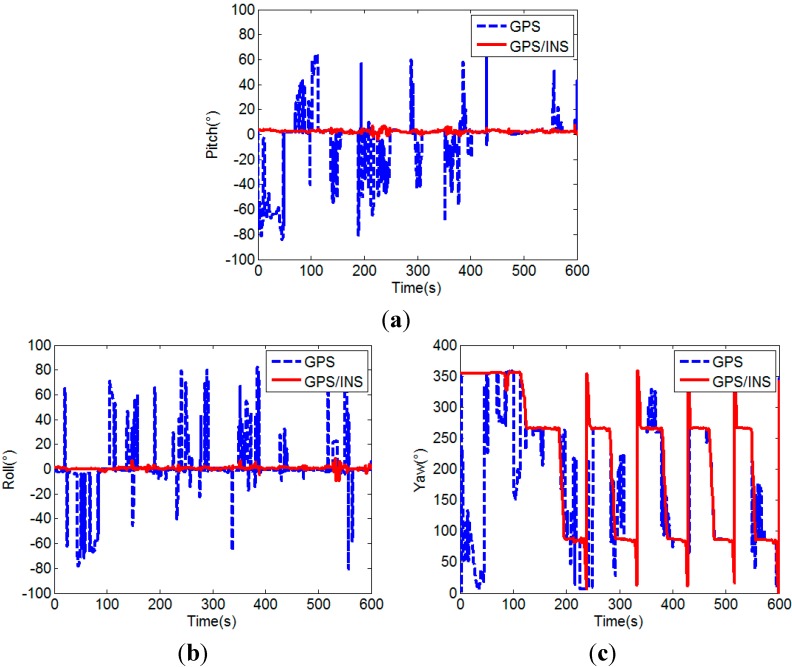
Comparison of the pitches (**a**), rolls (**b**) and yaws (**c**) of unaided GPS and MEMS-INS aided GPS.

The success rate of the proposed MEMS-INS aided GPS attitude determination method (GPS/INS) is analyzed by comparison with the unaided single frequency single epoch GPS attitude determination method (GPS). In the experiment, since the car moves on a flat road, the pitches of the two baselines can be considered constant in a certain allowable error range, here we use their initial values as the references and set 5° as the error range. The comparison of the attitudes calculated by the two attitude determination methods is shown in Figure 12.

From Figure 12, it can be seen that the attitude calculated by the MEMS-INS aided GPS method is more accurate and stable than that of the unaided GPS method. The yaw calculated by the MEMS-INS aided GPS method clearly shows the movement of the car. The lines approximately vertical to the angle axis represent the movement along a straight line, the two changing parts of the curve before 150 s show the starting and 90° turn to the rectangular block, and the dramatically changing parts of the curve after 150 s show the 180° turn of the car for the 5 laps. However, it can not be obtained by the attitude calculated by the unaided GPS method, because unsuccessful attitude determination often occurs in the unaided single frequency single epoch case, which results in large attitude error.

**Figure 13:**
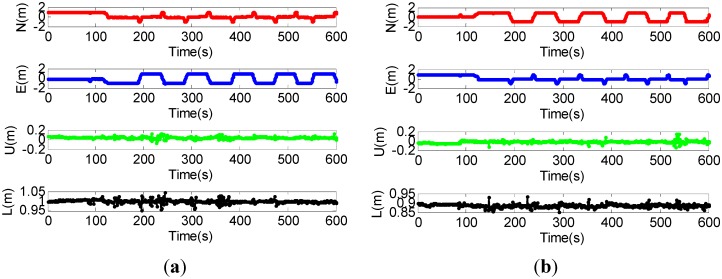
(**a**) Coordinates of the main baseline; (**b**) Coordinates of the auxiliary baseline.

Figure 13 shows the coordinates of baseline in the local frame of the MEMS-INS aided GPS method, where N, E, and U denote the north, east and up coordinates of baseline, respectively; L denotes the length of baseline. The maximum error of the baseline length is 0.05 m, which indicates that accurate baseline coordinates can also be obtained by the aided method.

Then, the success rates of the MEMS-INS aided GPS and the unaided GPS attitude determination methods are given in Table 1. The results can be expected after the float solution quality verification. Since external factors such as multipath and low satellite visibility in urban environment further degrade the measurement quality of the GPS receivers, the success rate of the unaided GPS attitude determination is not very high due to the low-quality float solution. The success rate of the proposed MEMS-INS aided GPS method is above 98%, which is much higher than that of the unaided method.

**Table 1:** Success rates of two methods.

	Unaided GPS	MEMS-INS Aided GPS
Main baseline	72.83%	99.00%
Auxiliary baseline	70.67%	98.83%

**Figure 14:**
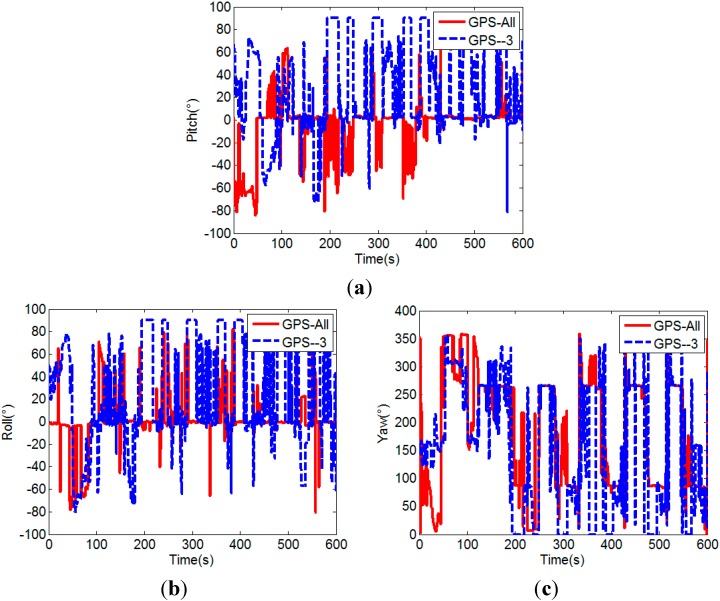
Pitches (**a**), rolls (**b**) and yaws (**c**) of unaided GPS method.

**Figure 15:**
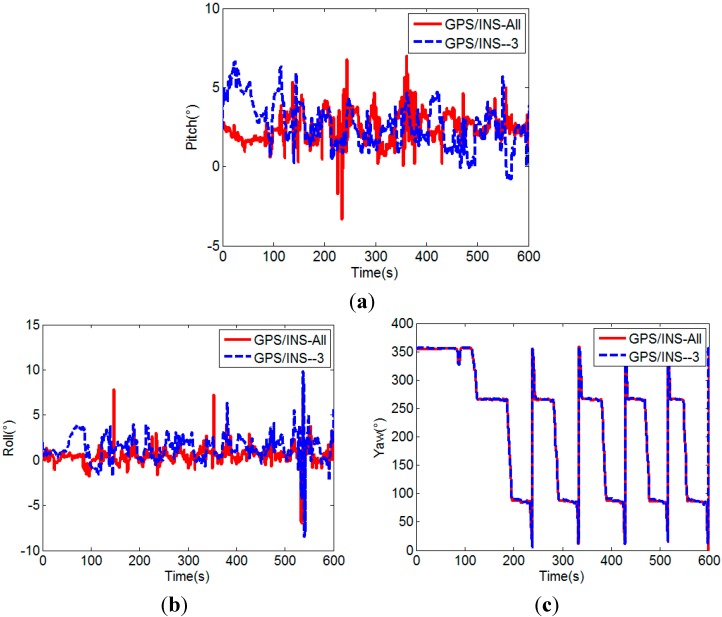
Pitches (**a**), rolls (**b**) and yaws (**c**) of MEMS-INS aided GPS method.

Figure 14 and Figure 15 further show the influence of the number of visible satellites on the attitudes calculated by the two attitude determination methods, respectively. When the number of visible satellites is less than four, the unaided GPS method cannot determine the attitude, so the pitch and roll are set to 90°, and yaw is set to 0° for drawing the figures. The corresponding success rates are listed in Table 2.

As shown in Figure 14 and Figure 15 and Table 2, the success rate of the unaided GPS method obviously decreases to below 50% after the three satellites with lowest elevation angles are removed, but for the MEMS-INS aided GPS method, the success rate slightly decreases about 1%, which in general is still very high (above 97%). Its advantage is very evident, since the attitude determination is also successful at times of two visible satellites.

**Table 2:** Success rates for different number of visible satellites.

	Unaided GPS (All/-3)	MEMS-INS Aided GPS (All/-3)
Main baseline	72.83%/49.50%	99.00%/97.83%
Auxiliary baseline	70.67%/45.67%	98.83%/97.67%

From the above results, it can be seen that the success rate of GPS/MEMS-INS attitude determination is usually unable to achieve 100%, since the maximum success rate depends on the quality of carrier phase measurement and the performance of MEMS-INS in the land vehicle application. When the integer ambiguity resolution is unsuccessful, correct attitude can not be obtained, and the attitude error may be large. It is no doubt that if the abnormal GPS attitude measurement is used in the integrated filter, the large error will degrade the overall attitude accuracy of the integrated system. To avoid this problem, a simple quality control (QC) method is adopted in the GPS/MEMS-INS integration, which is based on the residual chi-square test [6,10].

(3) Attitude accuracy verification

**Figure 16:**
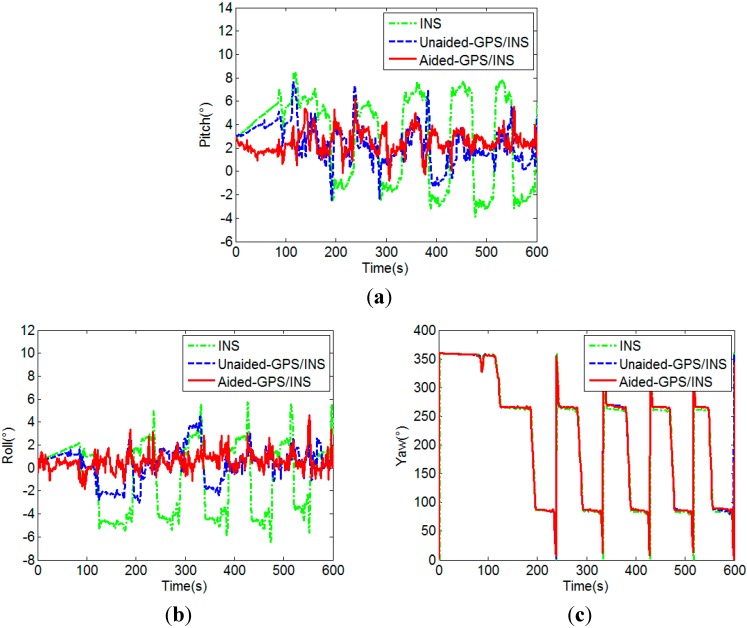
Pitches (**a**), rolls (**b**) and yaws (**c**) of MEMS-INS and the integrated navigation.

The final attitude results obtained from the integrated filter are shown in Figure 16, which contain the results of the unaided GPS/MEMS-INS integration and the aided GPS/MEMS-INS integration. For further analysis, the attitude results of stand-alone MEMS-INS are also given in Figure 16, whose initial value is assigned by GPS attitude determination. The standard deviations of pitch and roll are shown in Table 3.

**Table 3:** Standard deviations of the pitch and roll.

	MEMS-INS	Unaided Integration (All/-3)	Aided Integration (All/-3)
Pitch (°)	3.7920	1.9344/2.6892	1.0823/1.4216
Roll (°)	3.0991	1.5886/2.6534	1.0626/1.4209

As compared with Figure 12, Figure 16 demonstrates that the attitude accuracy is improved through the integration of GPS and MEMS-INS. It also demonstrates that the attitude results of the aided GPS/MEMS-INS integration are much better than that of the unaided integration. Since the success rate of MEMS-INS aided GPS attitude determination is much higher, the attitude errors of MEMS-INS can be corrected effectively by the GPS/MEMS-INS integration. It can be seen from Table 3 that the attitude accuracy improvement by the aided GPS/MEMS-INS integration is significant, especially for a GPS challenged environment. 

## 6. Conclusions

In order to improve the performance of the low-cost GPS/MEMS-INS attitude determination system used in land vehicles, a new integrated attitude determination method is presented. The core issue of the method is how to improve the success rate of the single frequency single epoch GPS ambiguity resolution with low-quality measurements. We adopt the GPS/INS measurements augmentation, the quality assessment of float solution and the undesirable float solution replacement by INS aided AFM to improve the quality of the float solution. Then, the CLAMBDA method is used to obtain the fixed solution, which can maximize the ambiguity success rate for attitude determination. Finally, the GPS/MEMS-INS integrated filter is designed to increase the attitude accuracy. Field test results in urban area demonstrate that our proposed method can significantly improve the quality of the float ambiguity solution, the success rate, and the accuracy of attitude determination especially for GPS challenged environment. The success rate increases to above 97%. Compared with the unaided GPS/MEMS-INS integration, the attitude accuracy is improved at least 35%. The improved low-cost GPS/MEMS-INS attitude determination system can offer a superior performance and efficiently fulfill the task in the land vehicle application.
